# Research note: the effects of providing a sprouted barley supplement to laying hens (*Gallus gallus domesticus*) on egg production parameters and feather cover

**DOI:** 10.1016/j.psj.2025.106161

**Published:** 2025-11-26

**Authors:** Victoria Sandilands, Farina Khattak, Jos G M Houdijk

**Affiliations:** Monogastric Science Research Centre, School of Veterinary Medicine and Biosciences, SRUC, King’s Buildings, West Mains Road, Edinburgh, EH9 3JG, UK

**Keywords:** Laying hen, Fibre, Sprouted barley, Egg production, Egg quality

## Abstract

Feed represents the primary cost in egg production systems. Although fibre comprises a small portion of poultry diets, it can play a crucial role in supporting gut health, improving litter quality, and reducing feather pecking. Sprouted grains, such as barley, provide a source of fibre along with additional nutritional benefits, yet their impact on laying hens' productivity and behaviour are not well documented. This study examined the effects of supplementing Lohmann’s Brown hen diets with hydroponically sprouted barley (**SB**), offered at 0, 15, 30, or 45g/hen/day over six weeks on body weight, egg production, egg quality, feed intake, and feather damage as an indicator of feather pecking. Each treatment had six replicate pens of 5 hens each. Data were analyzed for the effects of supplemental SB, time (i.e., study day, week, or period), and their interaction. Despite high palatability and complete SB consumption, no significant differences (P > 0.05) were observed in body weights, basal diet (BD) feed intake, egg production, egg weight, egg mass, most egg quality measures, or feather damage, although the highest level of SB tended to reduce BD intake while maintaining total dry matter intake. However, a significant effect of SB on eggshell strength was observed primarily due to an interaction between SB level and study day (*P* = 0.021). At day 21, eggs from hens on SB 15 and 45g/hen/day had reduced shell strength compared to those on SB 0 and 30, while at day 42, eggs from SB 0 hens showed greater shell strength than SB 15 hens. Feather damage worsened somewhat over the study period but was not related to SB treatment. In conclusion, most observed effects were attributed to the duration of the study rather than the level of SB inclusion. A larger-scale study using a single optimised SB level is warranted.

## Introduction

Egg production is a major global industry, producing over 87 million metric tonnes in 2022 (Shahbandeh, 2024). In the UK, free-range systems dominate the egg market, accounting for 60 % of all eggs processed through packing centers in 2023 (Defra, 2024). Irrespective of the production system, feed remains the single largest element of production costs, contributing almost 44 % of total expenses for free-range egg producers (ADAS, 2024).

Commercial layer diets are formulated to support optimal hen health and egg production, yet they typically contain low crude fiber levels (2.5–7 %), depending on ingredient composition and inclusion rates (Pottgüter, 2019). Although high fibre levels may be anti-nutritive, small amounts of dietary fiber are now recognised as beneficial for supporting gut health, improving litter quality and particularly with insoluble fibre, can help mitigating feather pecking behaviors (van Krimpen et al., 2012) which remain a persistent welfare concern in laying hens.

One approach to increasing fiber intake is to offer hens germinated, or sprouted, grains, since sprouting tends to increase the proportional fiber content, largely through the use of starch during germination (Singh et al., 2015). Hydroponic systems provide an efficient way to produce sprouted grains, using minimal water and no soil. Although fiber-rich diets have been linked to reduced feather pecking, it remains unclear whether hydroponically-sprouted barley, which shows increased crude fiber content during germination (Gebremedhin, Deasi and Mayekar, 2015), delivers comparable welfare benefits. Furthermore, the effects of sprouted barley on egg production, nutrient utilization, and feather condition in layers remain poorly defined. Most existing research on sprouted grains in poultry has focused on broilers, leaving a knowledge gap regarding their application in laying hen systems.

The aim of this study was to evaluate how providing hydroponically-sprouted barley supplements at different inclusion levels affected laying hen performance, including egg production, basal diet intake, indicators of feather pecking and egg quality.

## Materials and methods

This study was approved by SRUC’s Animal Welfare and Ethical Review Body (POU AE 23-2022).

### Animals and housing

Lohmann Brown hens were used in this experiment. At 42 weeks of age, 120 hens were transferred from enriched cages within the research facility (where they had been fed a commercial layer mash diet for 26 weeks) to the experimental room. Each hen was leg-ringed on arrival, and allocated to one of 24 litter-floor pens, with five hens per pen. Pen assignments ensured that hens remained with familiar conspecifics to minimize social disruption. Each 2 × 1 m pen was constructed with wood-and-wire walls (height: 3 m), allowing visual contact between adjacent pens. Each pen contained a nest box, an automatically-filled bell drinker, manually-filled feed hopper, galvanized feed trough (approximately 10 × 50 cm) for sprouted barley supplement, a perch (66 cm long), and a bundle of polypropylene string as a pecking enrichment. Throughout the study, all hens received the same commercial layer’s mash (basal diet; BD) with a calculated nutrient content of: metabolizable energy 11.56 kcal/kg; crude protein 17.48 %; crude fibre 2.90 %; calcium 3.84 % and available phosphorous 0.28 %. Hens were maintained on a 14 h light schedule (lights on: 05:00 to 19:00 h). Room temperature was controlled at 19°C (min 18.4, max 19.7) and relative humidity was maintained at an average of 74.2 % (min 71.0 %, max 79.2 %), with values recorded daily.

A four-week acclimatization period was provided before the start of the experiment, which commenced when the hens were 46 weeks of age. Sprouted barley treatments were randomly allocated into six blocks of four pens, based on their location within the facility (room block design). Each treatment was thus replicated across six pens (n = 6). Pens within each block were randomly assigned to one of the four treatments that provided 0, 15, 30 or 45 g/hen/day of hydroponically-sprouted barley (SB) for six weeks (days 0–42). At the end of the study (at day 42), all hens were humanely culled.

### Sprouted barley production

On the first day of hydroponic SB production (day 0), 3 kg of dry barley grain was weighed, rinsed, and soaked in tap water for approximately 8 hours. Then 1 kg of drained wet grain was evenly distributed onto each of four clean trays. Trays were placed on tables under LED lights (intensity 260–370 lux) from 06:00 to 21:00 h daily in a temperature-controlled room maintained at 23°C. Tray positions were rotated daily to ensure uniform light exposure.

The barley was wetted daily (either by rinsing in a colander under running water for 20 sec before replacing in the tray on days 1-4 or misting in the tray for 10-15 sec on days 5-6). SB was harvested on day 7 or 8, depending on root mat development; trays held to day 8 were sprayed with water again on day 7. Harvested SB was weighed and apportioned according to treatment, offered once each morning in galvanized feed troughs and any SB remaining after 6 h was weighed and recorded. To ensure continuous supply, the full SB production cycle was repeated daily. The determined composition of SB was 5.70 % crude fiber, 11.43 % crude protein, 1.70 % ash, and 51.30 % dry matter.

### Body weights and basal diet consumption

Hens were individually weighed on experimental days 0, 21 and 41. Basal diet (BD) was weighed into feed hoppers on day 0 and replenished as required for *ad libitum* access. On days 21 and 42, the residual BD in each hopper was weighed and recorded. BD intake was estimated by subtracting the remaining feed from the total BD offered. BD intake was calculated for the periods 0–21 days, 21–42 days, and cumulatively over 0–42 days.

### Egg production and egg quality

All eggs per pen were collected daily, and the total number of eggs was recorded to determine egg production:

$$\text{Egg}\;\text{production}\;\left(\%\right)\;=\left[\left(\frac{no\;of\;eggs\;collected\;in\;that\;period}{no\;of\;hens\;in\;that\;period\;}\right)*100\right]$$

Each egg was visually scored for absence/presence of external defects (e.g. dirt, cracks or shell-less). Only eggs without external defects (good eggs) were bulk weighed per pen per day to calculate egg mass (mean percentage of good eggs produced × mean good egg weight). Feed conversion ratio (FCR: BD consumption/good egg weight), was assessed per pen for each period (0-21 days, 21-42 days, and cumulatively over 0-42 days). On day 0, 21, and 42, all good eggs were further tested for egg quality parameters, including egg weight (g), albumen height (mm), yolk colour (YolkFan^TM^ colour scale 1-16), Haugh units, shell strength (kgf), shell thickness (mm), yolk height (mm), and yolk diameter (mm) using a Nabel Digital Egg Tester (model DET-6500, Kyoto, Japan).

#### Feather damage

On day 0, 21 and 41, each hen was assessed for observable damage to feathers and/or skin at seven body locations (breast, neck, back and rump, belly and flank, tail, left wing, right wing). Each location was scored from 0 (no damage) to 5 (1-2 cm^2^ haemorrhage, or >5 × 5 cm^2^ bare skin, with <1 cm^2^ haemorrhage). A total feather damage score was calculated by summing all scores per hen.

### Statistical Analysis

Data were initially processed in Excel and then imported to Genstat (version 23.1, VSN International Ltd.) for statistical analysis. One pen was excluded due to consistently low body weights and egg production, therefore calculations were performed on n = 5 replicates for treatment SB 45. Data collection was over 42 days (days 0-42), therefore day 0-7 was classed as week 1. Data were analyzed at the pen level for effects of SB treatment (0, 15, 30, 45 g/hen/d) and time period on: BD intake (day 0-21, 21-42, and day 0-42), egg quality measures (day 0, 21, and 42, after mean values per pen were calculated for each day), FCR (day 0-21, 21-42, and day 0-42); egg production (%), egg weight (g), egg mass (kg) were analyzed weekly, after mean values per pen per week were calculated from daily values. Egg defects were not analyzed due to low numbers. Body weights and feather damage (both measured on days 0, 21, and 41) were analyzed at the bird level for effects of SB treatment and day.

BD intake and FCR were analyzed by ANOVA with pen as block, including effects of SB level, feed period (0-21, and 21-42 days), and their interaction. BD intake was further analysed by ANOVA using orthogonal polynomials to investigate linear or quadratic relationships between SB offered and BD intake. SB intake was not analyzed because hens consumed the full allocation after week 2. Egg production, egg weight, egg mass, and egg quality measures were analyzed by repeated measures ANOVA, with pen as block, and week as time effect. Body weights and feather damage were analysed using Generalised Linear Mixed Model (GLMM), with a poisson distribution and logarithm link function, with bird, pen and room block as random effects and SB treatment, day and treatment.day as fixed effects.

Data were considered significantly different at P ≤ 0.05. Variation is expressed as standard deviation (SD) or standard error of means (SEM).

## Results and discussion

One hen in SB15 was culled during the acclimatisation period due to an enlarged crop and one hen in SB0 was found dead on day 35, with no obvious cause.

### Body weights, SB intake and BD intake

Mean body weights of hens were similar across treatments at the start of the experiment (day 0), and at subsequent weighing days on day 21 and 41 (range across all treatments and days: 1.822-1.901 kg). There were no significant effects of SB treatment (P = 0.829, SEM 0.023), day (P = 0.760, SEM 0.012) or their interaction (P = 0.650, SEM 0.024) on hen body weights.

SB intake was high from the outset, with only 0.3-2.4 g left on average per treatment in week 1, and complete daily consumption from week 3 onwards, indicating good palatability. BD intake ranged from 134 to 172 g/hen/d during 0-21 days, and 100-136 g/hen/d during 21-42 days. Contrary to expectations, BD intake was not significantly reduced by SB treatment (P = 0.188, SEM 11.8) but was significantly higher during the first feeding period (day 0 to 21; x̄=150 g/hen/d) than during the second (day 21 to 42; x̄=123 g/hen/d) (P < 0.001, SEM 4.5). There was no treatment by study period interaction (P = 0.368, SEM 13.6), nor a treatment effect on overall BD intake (days 0-42) (P = 0.190, SEM 12.0). However, a linear trend was present on overall BD intake (P = 0.065), driven by a reduction in BD intake in SB45 vs SB0 (x̄=116 vs 142 g/hen/day, respectively). When total dry matter intake (from BD and SB) was considered, intake was similar for SB0 and SB45 (x̄=125 and 126 g/hen/d, respectively), indicating that hens compensated for higher SB provision by reducing BD consumption. Comparable reductions in basal diet intake with provision of barley fibre have been reported by Raginski et al. (2023).

#### FCR

FCR was not affected by either SB treatment (P = 0.293, SEM 0.120) or the interaction of SB treatment with period (P = 0.326, SEM 0.227) with mean values ranging from 1.880 to 2.994. FCR was significantly lower during day 21 to 42 (x̄= 2.239), driven largely by the lower value for SB45 (1.880), compared with days 0 to 21 (x̄= 2.645) (P = 0.001, SEM 0.077). Over the entire study (day 0 to 42), SB had no effect on FCR (P = 0.287, SEM 0.201).

### Egg production

**Fig. 1:**
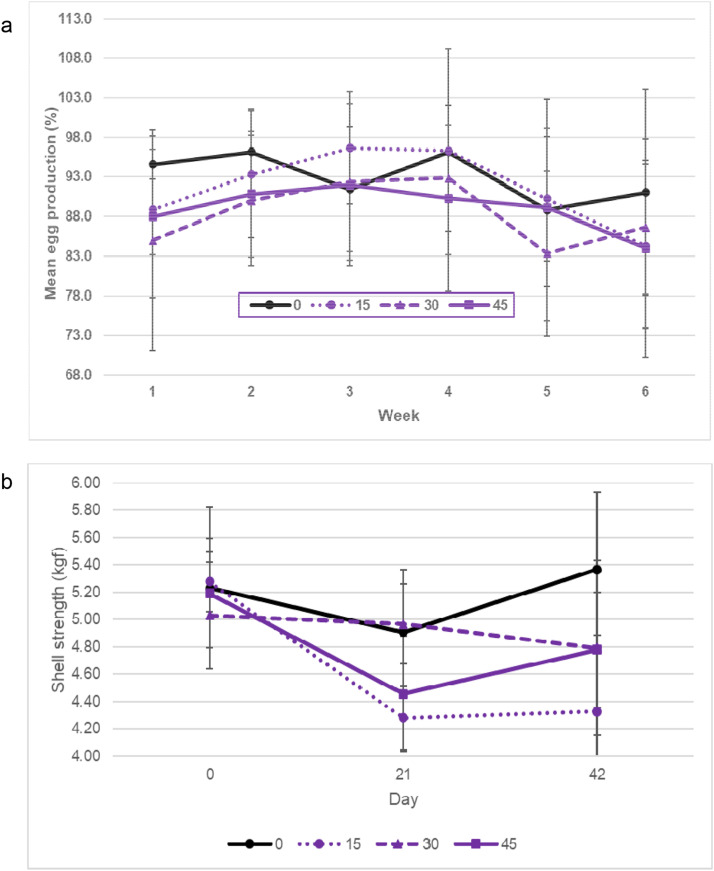
a) Mean egg production (%) with standard deviation bars shown, for each treatment by study week (1-6); b) Mean shell strength (kgf) with standard deviation bars shown, for each treatment by study day (0, 21, 42). Treatments were sprouted barley (SB) fed at 0, 15, 30, or 45 g/hen/day. N = 6 pens/treatment (5 hens/pen), apart from SB treatment 45, where n = 5 pens.

Of 4,434 total eggs collected, only 16 eggs had external defects, therefore, egg defects were not analyzed. Across treatments and weeks, egg production ranged from 96.7 % to 83.3 % (Figure 1a) and was affected by week (P = 0.015, SEM 0.016), consistent with previous studies (e.g. Tainika et al., 2024). There was no significant effect of SB treatment (P = 0.562, SEM 0.026) or SB treatment by week interaction (P = 0.828, SEM 0.039).

Egg weights ranged from 60.3 to 62.5 g and were unaffected by treatment (P = 0.865, SEM 0.0006), week (P = 0.138, SEM 0.0002), or their interaction (P = 0.546, SEM 0.0007). Mean egg mass (kg)/hen/day was significantly affected by week (P = 0.030, SEM 0.0011), but not by SB treatment (P = 0.697, SEM 0.0017) or their interaction (P = 0.844, SEM 0.0026). Mean egg mass/hen/day was highest in week 4 (0.0575 kg), and lowest in weeks 1 (0.0540 kg) and 6 (0.0529 kg), with intermediate values in weeks 2 (0.0568) 3 (0.0571) and 5 (0.0541). These results indicate no adverse effects of SB levels on these measures.

#### Egg quality

Both albumen height and Haugh unit were significantly affected by study day (P < 0.001, Table 1) with significantly lower values on day 0 than on days 21 and 42. Shell strength was significantly affected by treatment (P = 0.049), day (P < 0.001), and their interaction (P = 0.021, Figure 1b). Starting from similar shell strength at day 0, only SB0 maintained a consistent shell strength throughout the trial. By day 21, SB15 had the weakest shells, although this did not differ significantly from SB45. By day 42, eggs from hens fed SB at any inclusion level had significantly weaker shells than those from SB0 hens. Despite this, all shell strength levels across treatments exceeded acceptable ranges (e.g. 3.5-4.5 kgf) for table eggs (Kashimori, 2017). It may be that, as sprouting increases phytase, which may enhance release of phytate-bound minerals including phosphorus and calcium (Elliott et al., 2022), SB treatments could increase the bio-availability of minerals, thus improving shell strength, provided minerals are correctly balanced in the total intake (BD + SB). That balance may have been distorted here, potentially resulting in weaker shells. Future studies on SB supplementation responses to egg quality, including shell strength and thickness, may benefit from assessing calcium retention to clarify these potential interactions. There was no effect of SB treatment, day, or their interaction on shell thickness, egg weight, yolk colour, yolk diameter, or yolk height.

**Table 1:** Mean egg quality measurements according to SB treatment (0, 15, 30, 45 g/hen/d), day (0, 21, 42) and their interaction^1^.

**Egg quality parameters**	Albumen height (mm) (P)	Haugh unit (HU)	Shell strength (kgf)	Shell thickness (mm)	Egg weight (g)	Yolk colour	Yolk diameter (mm)	Yolk height (mm)
Treatment								
0	7.4	85.0	5.2^a^	0.4	61.5	5.5	40.3	17.5
15	7.4	83.7	4.6^b^	0.4	61.5	5.5	42.7	17.8
30	7.3	84.3	4.9^ab^	0.4	61.1	5.5	41.2	17.6
45	7.7	87.6	4.8^b^	0.4	59.7	5.0	41.5	17.5
*P*-value	0.514	0.142	0.049	0.584	0.524	0.294	0.611	0.718
SEM	0.20	1.20	0.13	0.01	0.95	0.20	1.25	0.22
Day								
0	6.7^b^	79.9^b^	5.2^a^	0.4	60.3	5.6	40.4	17.5
21	7.9^a^	87.7^a^	4.7^b^	0.4	61.4	5.4	42.7	17.7
42	7.8^a^	87.8^a^	4.8^b^	0.4	61.2	5.2	41.4	17.6
*P*-value	<0.001	<0.001	<0.001	0.794	0.223	0.146	0.161	0.279
SEM	0.17	1.25	0.08	0.00	0.46	0.15	0.82	0.10
Day × Treatment								
*P*-value	0.157	0.088	0.021	0.676	0.160	0.810	0.563	0.448
SEM	0.34	2.37	0.18	0.01	1.22	0.32	1.83	0.28

1 Where superscripts are different in a column, by factor, means are significantly different (*P* < 0.05). N = 6 pens/treatment (5 hens/pen), apart from SB treatment 45, where n = 5 pens.

#### Feather damage

Feather scores for individual body location did not exceed 2.5, indicating good feather cover and thus low levels of feather pecking behaviour. Mean feather scores by SB treatment ranged from 5.8 to 7.2 on Day 0, 7.1-8.4 on Day 21, and 8.0-9.2 on Day 41. Feather damage was affected by day (P < 0.001, SEM 0.042) but not by SB treatment (P = 0.527, SEM 0.081) or their interaction (P = 0.650, SEM 0.085). The gradual increase in feather scores over time aligns with expectations as hens age. (e.g., Freire, Walker and Nicol, 1999). Access to litter for foraging, a factor known to reduce feather pecking (Schreiter et al., 2019), likely contributed to the low incidence observed here. Because feather pecking did not occur, the study could not assess whether SB supplementation would help mitigate this behaviour. Fibre provided through SB may reduce feather pecking (van Krimpen et al., 2012; Desbruslais et al., 2021), but this requires the behaviour to be present.

Future research should explore the mechanisms by which soluble fiber affects eggshell integrity, particularly focusing on calcium absorption. Additionally, studies with larger sample sizes, focusing on the preferred inclusion level of 30 g SB/hen/d, would help validate these findings especially when SB is supplemented alongside a commercial mash diet. Although SB may offer nutritional benefits and temporarily boost egg production, its use should be considered carefully in relation to the overall mineral and nutrient balance in the diet. Longer term studies are needed to determine whether fresh SB supplementation supports sustained hen health and productivity, and to clarify any potential effects on feather pecking behaviour.

## CRediT authorship contribution statement

**Victoria Sandilands:** Conceptualization, Formal analysis, Funding acquisition, Methodology, Writing – original draft, Writing – review & editing. **Farina Khattak:** Data curation, Methodology, Writing – review & editing, Project administration. **Jos G M Houdijk:** Formal analysis, Methodology, Writing – review & editing.

## Disclosures

The authors declare the following financial interests/personal relationships which may be considered as potential competing interests: JGMH has established a working relationship with a local farmer who uses sprouted barley for his hen flocks. This in part stimulated our desire to research the effects of sprouted barley on egg production parameters and hen welfare. If there are other authors, they declare that they have no known competing financial interests or personal relationships that could have appeared to influence the work reported in this paper.
